# Alterations in aortic elasticity indices among type 2 diabetes patients in a low and middle income country using M-mode echocardiography: A cross-sectional comparative study

**DOI:** 10.1371/journal.pone.0305799

**Published:** 2024-10-24

**Authors:** Hai Nguyen Ngoc Dang, Thang Viet Luong, Quan Nguyen Khoi, Uyen Ngoc Phuong Nguyen, Nguyen Nguyen Khoi Pham, Hieu Thi Nguyen Tran, Hung Khanh Tran, Mai Thi Thu Cao, Binh Anh Ho, Thang Chi Doan, Hung Minh Nguyen, Tien Anh Hoang, Minh Van Huynh

**Affiliations:** 1 The Faculty of Medicine, Duy Tan University, Da Nang, Vietnam; 2 University of Medicine and Pharmacy, Hue University, Hue, Vietnam; 3 College of Health Sciences, Vin University, Hanoi, Vietnam; 4 Johns Hopkins University Bloomberg School of Public Health, Baltimore, MD, United States of America; 5 Cardiovascular Center, Hue Central Hospital, Hue, Vietnam; 6 Vietnam National Heart Institute, Bach Mai Hospital, Ha Noi, Vietnam; University of Pisa, ITALY

## Abstract

**Background:**

Diabetes is one of the leading causes of noncommunicable diseases worldwide. It is known to induce cardiovascular remodeling, which can result in a variety of complications, including a considerable increase in aortic stiffness. While studies in Western populations have explored these effects, data on Asians, mainly Vietnamese, are limited. This study aimed to assess aortic elasticity in type 2 diabetes mellitus (T2DM) patients compared to healthy individuals.

**Methods:**

This quantitative, cross-sectional study compared aortic elasticity indices between individuals with T2DM and healthy controls in Vietnam. Aortic elasticity indices were assessed for all participants using M-mode echocardiography.

**Results:**

A comparison between the healthy and T2DM groups revealed substantial differences in aortic elasticity indices. The aortic stiffness index (ASI) was significantly greater in the T2DM group than in the control group, with median values of 6.10 (3.64–12.47) and 3.79 (2.40–8.50), respectively (p = 0.003). Aortic strain (AS) was substantially lower in the T2DM group than in the control group, with median values of 8.21% (4.24–13.07) and 10.66% (6.01–18.23), respectively (p = 0.039). Furthermore, the median aortic compliance (AC, 10^-2^mm/mmHg) and aortic distensibility (AD, 10^-3^mmHg^-1^) in individuals with T2DM were 4.07 (2.28–7.44) and 3.08 (1.57–5.26), respectively, lower than those in the control group, with median values of 6.40 (3.08–10.75) and 5.33 (2.80–9.79). A longer diabetes duration was linked to a greater ASI (r = 0.43, p < 0.05), while the AS decreased (r = -0.37, p < 0.05).

**Conclusions:**

Substantial variations in aorta elasticity indices were found in patients with T2DM using M-mode echocardiography. These differences highlight the impact of T2DM on vascular health. More research is needed to investigate the consequences of these discrepancies and their significance for clinical purposes.

## Introduction

Type 2 diabetes mellitus (T2DM) is currently an emerging noncommunicable illness worldwide. According to the International Diabetes Federation (IDF), the number of individuals with T2DM is expected to increase globally, with 10.5% of people aged 20 to 75 years being diagnosed with diabetes by 2021. By 2045, this figure will increase by 46%, with an estimated 783 million people living with diabetes. Diabetes caused 2.3 million fatalities in the Western Pacific region in 2021, the highest among all IDF regions. Among these, Vietnam has almost 57 thousand diabetes-related fatalities in adults aged 20 to 79 years [1].

Many studies have shown that T2DM patients experience cardiac morphology and function changes over time after being diagnosed [2, 3]. In addition to damaging the heart, vascular remodeling has been shown to occur in patients with diabetes patients. Numerous studies on diabetes mellitus have revealed a strong link between vascular remodeling and the disease, influenced by various factors [4–6]. The most critical factors are insulin resistance and dyslipidemia [7]. Furthermore, because diabetes mellitus involves the risk of persistent hyperglycemia, glucose creates covalent connections with proteins, nucleic acids, and lipids through nonenzymatic glycosylation, also known as glycation. This irreversible process results in advanced glycation end products (AGEs). These chemicals accumulate in the vascular wall and, with time, impair the flexibility of blood vessels [8]. As diabetes progresses, patients are more likely to develop various vascular complications [5]. The duration of diabetes is associated with a higher risk of major cardiovascular diseases [9].

Several invasive and noninvasive procedures are available for measuring vascular elasticity [10–13]. Notably, noninvasive imaging techniques such as ultrasound, computed tomography and magnetic resonance imaging have emerged as effective methods for measuring arterial elasticity [14, 15]. Among these approaches, ultrasound stands out for its low cost, ubiquitous availability, and broad applicability, making it the recommended tool for measuring arterial elasticity.

Aortic elasticity, a measure of arterial stiffness, has been demonstrated to be a strong independent predictor of atherosclerosis and other cardiovascular events. This shows that measuring aortic flexibility could help detect vascular issues in their early stages [16]. However, most previous studies have concentrated on Western or Caucasian populations [17, 18]. There is a considerable knowledge gap about how aortic elasticity may change in people with T2DM in populations of low- and middle-income countries, specifically in the population of Vietnam. This study investigated the indications of aortic elasticity in T2DM patients and compared the aorta elasticity indices of T2DM patients and healthy control participants to determine patterns of change. By analyzing these parameters, this study aimed to gain valuable insights into how T2DM affects cardiovascular health, especially aortic elasticity, and contributes to an effort to detect and reduce disease burdens globally early.

## Methods

### Study population

**Fig 1:**
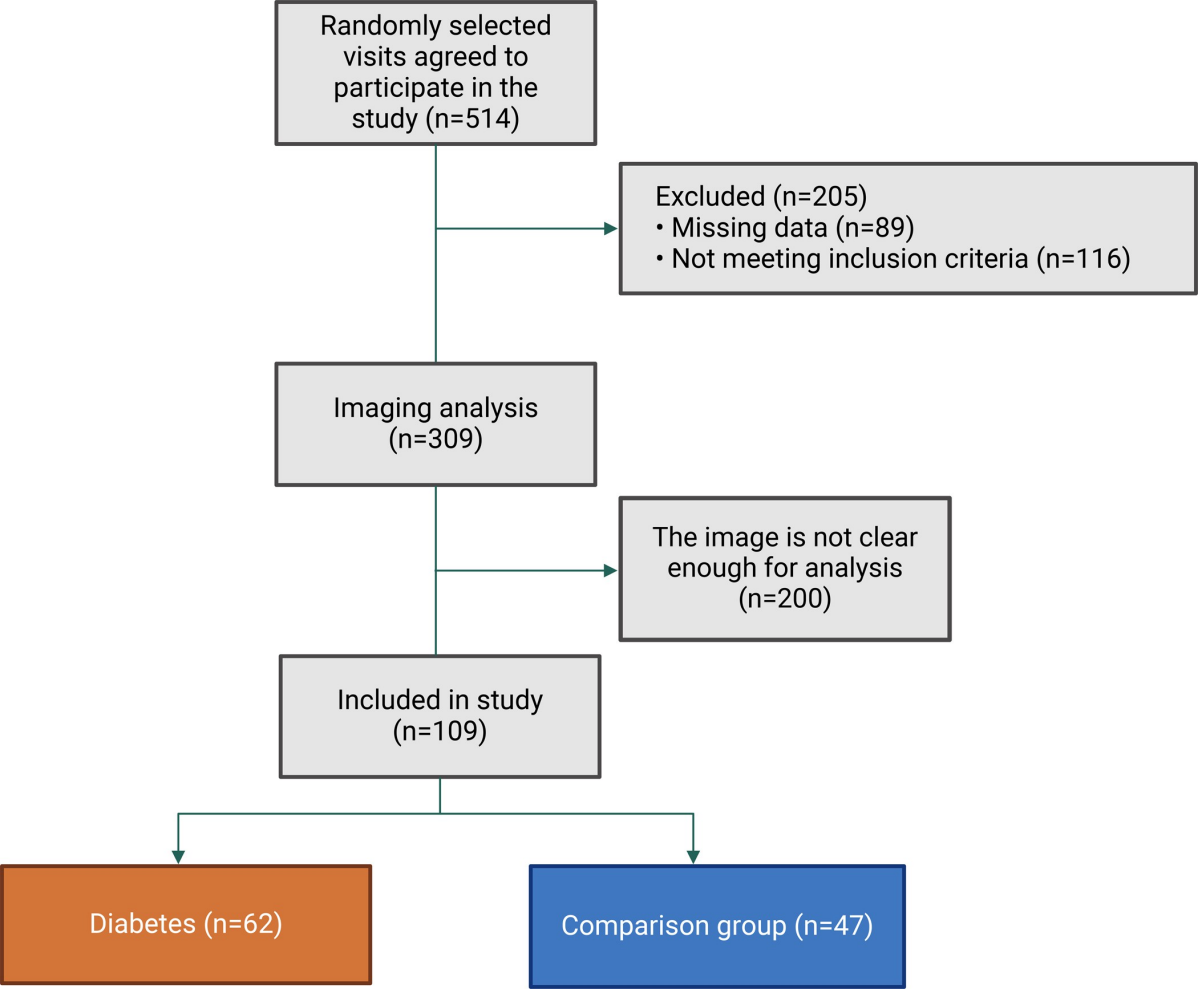
Flowchart of patient selection and data processing.

This is a quantitative, comparative cross-sectional study with a comparison group. We integrated components from the STROBE declaration to enhance the quality of reporting observational studies [19]. The study was conducted on 514 patients who agreed to participate at Hue University of Medicine and Pharmacy from 15/04/2022 to 01/06/2023. After excluding 89 subjects due to missing data and 116 based on exclusion criteria, 309 subjects were selected for ultrasound image analysis. Of these, 109 participants had analyzable images. The control group consisted of 47 healthy individuals who underwent regular health check-ups, had no history of diabetes or cardiac pathology, and were currently free from any cardiac or diabetic conditions. The remaining 62 participants were diagnosed with T2DM and had no known comorbidities, according to the diagnostic criteria stipulated by the American Diabetes Association in 2022 [20]. The detailed patient selection and data handling process is presented in **Fig 1**. The study received approval from The Institutional Ethics Committee of Hue University of Medicine and Pharmacy (Approval number: H2022/025) and was conducted under the principles outlined in the Declaration of Helsinki 2013. Before participation, written informed consent was obtained from each patient or their legal representative.

To ensure the validity of our analysis, we excluded patients who refused to participate and those with known comorbidities, such as hypertension. We also excluded patients with any cardiovascular disease, including severe aortic valve stenosis, moderate to severe aortic valve regurgitation, severe mitral valve stenosis, congenital heart disease, cardiomyopathy, myocardial ischemia, reduced ejection fraction, arrhythmia, or chronic atrial fibrillation. Additionally, patients with pacemakers, pregnant or breastfeeding women, individuals with acute life-threatening medical conditions, and those with poor or inadequate ultrasound images were excluded.

### Data collection

The data were collected using an ultrasound machine, paper medical records, and data collection forms to extract information from the study subjects. This process involved the following steps:

### Clinical examination and blood tests

After consenting to participate in this study, the patient’s medical history was obtained, emphasizing on characteristics such as the time of T2DM diagnosis and blood sugar management at each visit.

Body mass index (BMI) was computed using the following formula: BMI = weight (kg)/[height (m) × height (m)]. Body surface area (BSA) was calculated using the formula of Du Bois [21]: BSA = 0.007184 × (weight)^0.425^ × (height)^0.725^. Blood pressure was measured following the guidelines of the American Heart Association in 2019 [22, 23].

Blood samples for biochemical analysis were collected from fasting venous blood after waking. Biochemical tests were performed on a Roche Cobas E601 automated clinical chemistry analyzer at the Central Testing Unit of Hue University of Medicine and Pharmacy Hospital.

### Transthoracic echocardiography

Our study utilized a specialized Affiniti 70 echocardiography machine from Philips, the Netherlands, with the ability to perform time-motion mode (M-mode), two-dimensional mode, continuous-wave Doppler, color Doppler, and tissue Doppler imaging. The ultrasound machine simultaneously recorded the electrocardiography data along with the echocardiographic images. Transthoracic echocardiography was performed following the American Society of Echocardiography guidelines for adults [24].

### Arterial stiffness indices

M-mode echocardiography recorded the diameter of the ascending aorta 3 cm above the aorta valve in a parasternal long-axis image.

**Fig 2:**
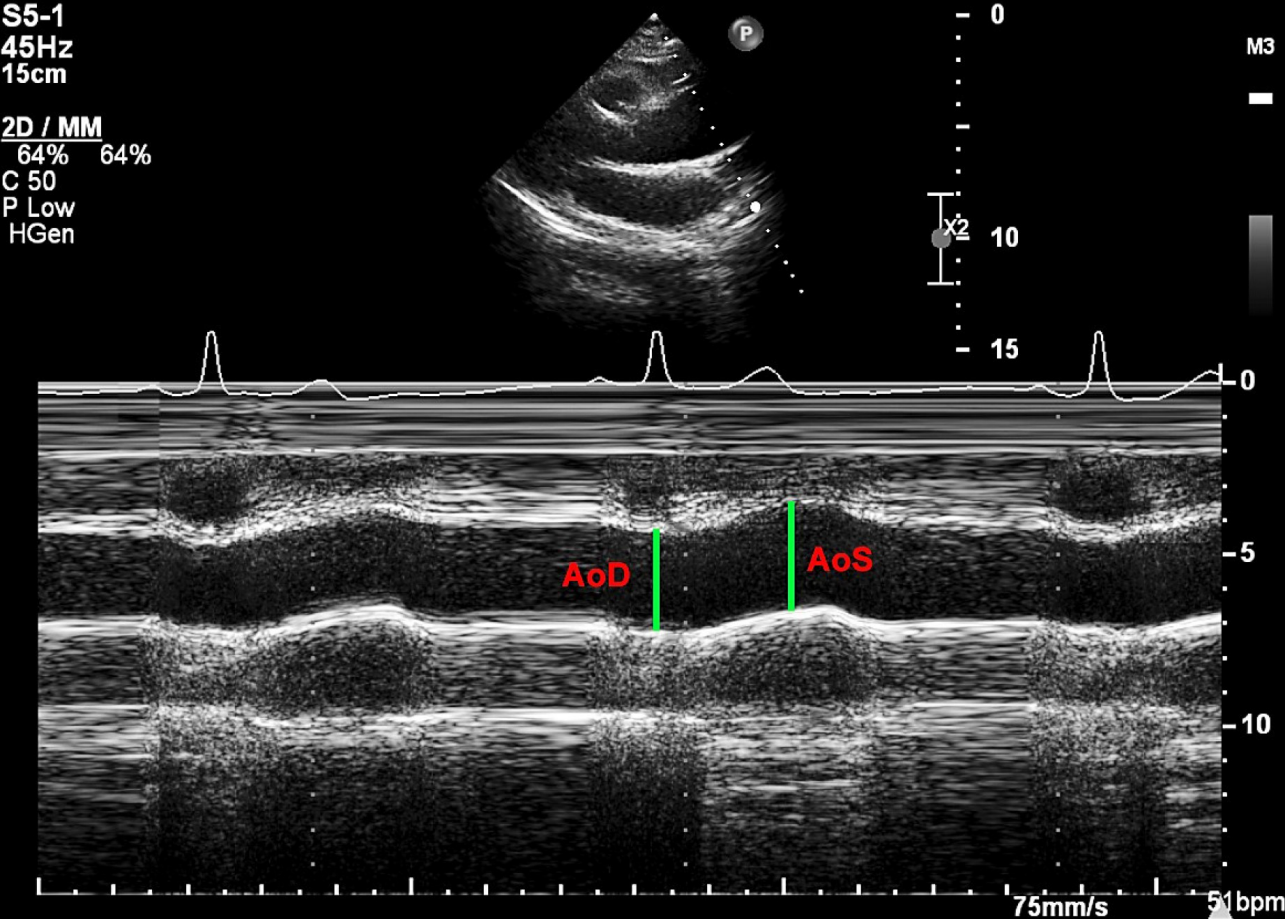
The aorta was visualized in M-mode. AoD: Diastolic diameter of the ascending aorta; AoS: Systolic diameter of the ascending aorta.

According to the guidelines provided by the American College of Cardiology, the American Heart Association, and the European Association of Cardiovascular Imaging for echocardiography in aortic diseases, the diastolic diameter of the ascending aorta (AoD) was measured at the peak of the R wave on the ECG. In contrast the systolic diameter of the ascending aorta (AoS) was measured at the point of maximum anterior motion of the aorta [25, 26]. The leading edge-to-leading edge method is recommended for measuring the ascending aorta (see **Fig 2**) [27, 28]. The diameter of the ascending aorta was measured in centimeters (cm). Additionally, systolic blood pressure (SBP) and diastolic blood pressure (DBP) refer to brachial systolic and diastolic BP, respectively, in millimeters of mercury (mmHg).

Aortic elasticity indices were calculated using the formulae below: aortic strain (AS) % = 100 x (AoS–AoD)/AoD, aortic stiffness index (ASI) = ln (SBP/DBP)/[(AoS–AoD)/AoD], aortic compliance (AC) mm/mmHg = (AoS–AoD)/(SBP–DBP), aortic distensibility (AD) mmHg^−1^ = [2 x (AoS–AoD)]/[AoD x (SBP–DBP)], and aortic Peterson’s elastic modulus (AEM) mmHg = (SBP–DBP)/[(AoS–AoD/AoD)]. Note: AoS refers to the aortic systolic diameter; AoD denotes the aortic diastolic diameter; SBP represents systolic blood pressure; DBP stands for diastolic blood pressure [29–31].

### Statistical analysis

The statistical analysis used SPSS software version 26 (IBM, Armonk, NY, USA). The Kolmogorov‒Smirnov test was used to determine the normality of the data. Normally distributed continuous variables are reported as the means ± standard deviations. Variables not normally distributed are represented as medians with interquartile ranges (IQRs = 25th– 75^th^ percentiles). Categorical variables are represented as frequencies and percentages. Missing data were omitted from the analyses. Individuals with and without T2DM were compared based on their baseline characteristics. Continuous variables were analyzed using independent t tests, and the results are reported as the means ± standard deviations. The Mann‒Whitney nonparametric test was applied to analyze variables that were not normally distributed. Categorical variables are presented as n (%) and were compared using the chi-squared test.

To compare arterial elastic parameters between the T2DM and non-T2DM groups, the Mann‒Whitney test was used for nonnormally distributed continuous variables. Spearman’s correlation coefficient (ρ) and P value elucidated the correlation between continuous variables. To determine whether the differences in aortic elasticity indices between the T2DM and non-T2DM groups were independent of blood pressure, we employed Generalized linear models (GLM). In the model, aortic elasticity indices served as the dependent variable, with group status (T2DM vs healthy control) as the primary independent factor. SBP and DBP were included as covariates to control for potential confounding.

All probability values were two-sided, and P values less than 0.05 were considered to indicate statistical significance. We followed the SAMPL guidelines in our statistical analysis to prevent avoidable errors or omissions in reporting statistical data [32].

## Results

### Clinical and echocardiographic features

This study included 109 subjects, including 62 T2DM patients and 47 healthy individuals who met the diagnostic criteria and composed the comparison group. Our research revealed the following results.

There were no significant differences in age, sex, height, weight, BMI, or BSA between the groups (p > 0.05). The differences in the left atrial diameter (LAd), left ventricular mass index (LVMI), and ejection fraction (EF) between the T2DM group and control group were not statistically significant (p > 0.05). Detailed information regarding these indices is presented in **Table 1**.

**Table 1:** Anthropometric and echocardiographic characteristics of participants by group.

Characteristics	T2DM group (n = 62)	Comparison group (n = 47)	P value
**Age (years)**	61.63 ± 10.46	60.40 ± 7.08	0.491
**Female (%)**	40 (64.5%)	26 (55.3%)	0.561
**BMI (kg/m** ^**2**^ **)**	22.26 ± 3.06	22.49 ± 2.35	0.657
**BSA (m** ^**2**^ **)**	1.53 ± 0.15	1.56 ± 0.13	0.300
**HbA1C (%)**	10.92 ± 3.02	5.03 ± 0.38	<0.001
**Duration of diabetes (years)**	6.00 [4.00–14.25]	NA	
**SBP (mmHg)**	127.63 ± 8.85	115.1 ± 12.19	<0.001
**DBP (mmHg)**	72.63 ± 7.02	72.32 ± 7.59	0.824
**LAd (cm)**	3.15 ± 0.42	3.19 ± 0.45	0.645
**LVMI (g/m** ^**2**^ **)**	107.31 ± 30.39	103.58 ± 23.08	0.488
**LVEF (%)**	67.92 ± 7.53	70.23 ± 5.27	0.075
**Total cholesterol (mmol/L)**	4.97 [3.63–6.49]	4.98 [4.15–5.00]	0.503
**Triglyceride (mmol/L)**	1.85 [1.06–2.95]	1.20 [1.05–1.25]	0.001
**HDL-C (mmol/L)**	0.96 [0.81–1.22]	1.25 [1.22–1.30]	< 0.001
**LDL-C (mmol/L)**	3.13 [1.96–3.67]	3.45 [2.73–3.55]	0.264

The values are presented as the means ± standard deviations, medians [Q25—Q75] or numbers (%). T2DM: type 2 diabetes mellitus; BSA: body surface area; BMI: body mass index; SBP: systolic blood pressure; DBP: diastolic blood pressure; LAd: left atrial diameter: LVMI: left ventricular mass index; LVEF: left ventricular ejection fraction; HbA1c: glycated hemoglobin; HDL-C: high-density lipoprotein cholesterol; LDL-C: low-density lipoprotein cholesterol.

### Aortic elasticity indices in the T2DM and control groups

**Fig 3:**
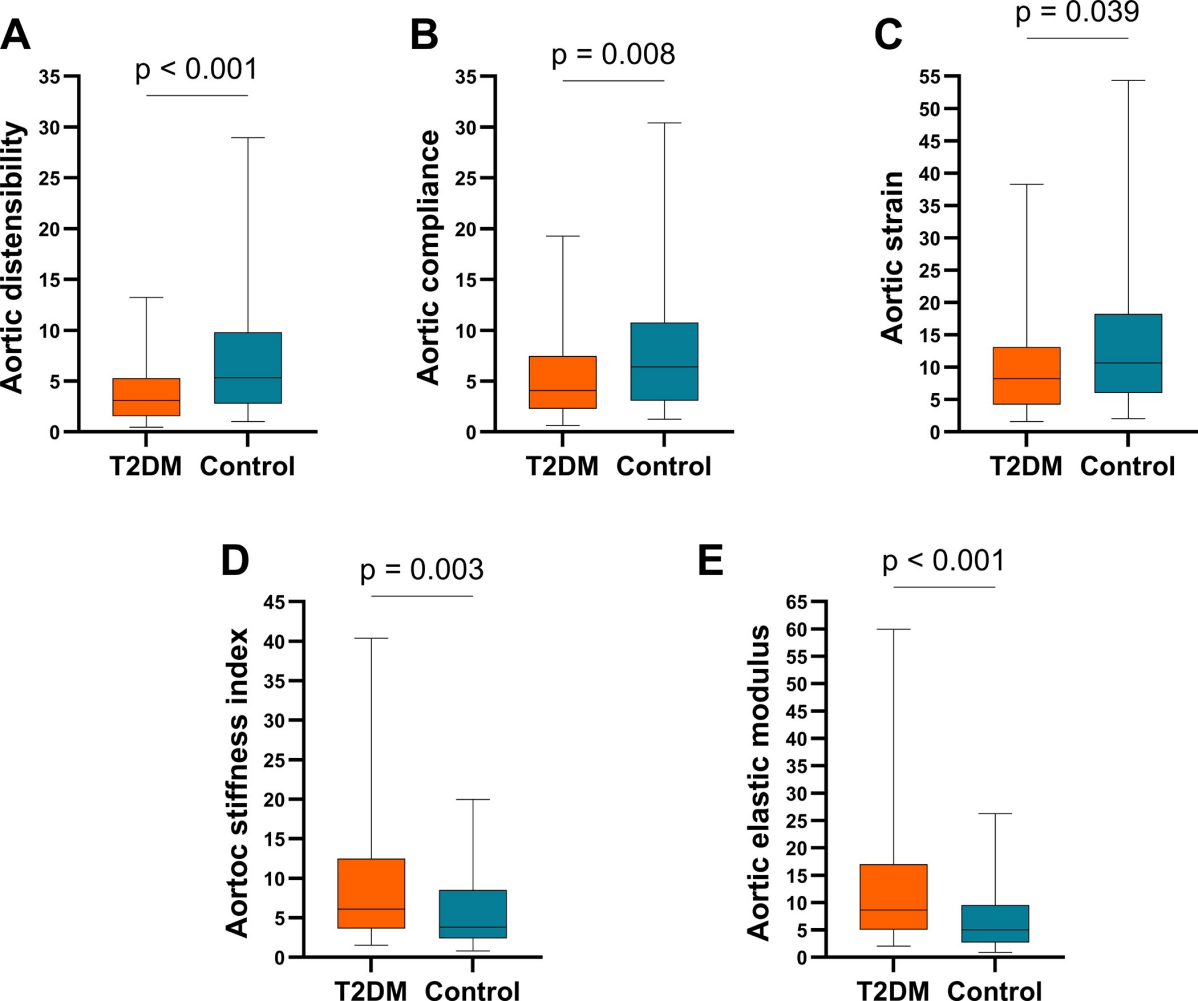
Comparison of aortic elasticity indices. (A) Comparison of aortic distensibility between the diabetes group and control group. (B) Comparison of aortic compliance between the diabetes group and the control group. (C) Comparison of strains between the diabetes group and control group. (D) Comparison of the aortic stiffness indices between the diabetes group and the control group. (E) Comparison of Peterson’s elastic modulus between the diabetes group and control group. T2DM: diabetes mellitus.

The T2DM group exhibited significantly lower AC, AD, and AS values than the control group. Conversely, T2DM patients demonstrated significantly higher ASI and AEM than the controls (p < 0.05). Detailed information is provided in **Table 2** and **Fig 3**.

**Table 2:** Computed aortic elasticity parameters of participants by group.

Arterial elastic parameters	T2DM group (n = 62)	Comparison group (n = 47)	P values
**AoS (mm)**	31.20 ± 4.95	27.08 ± 4.67	< 0.001
**AoD (mm)**	28.40 ± 4.32	24.04 ± 4.97	< 0.001
**AC (10** ^**-2**^ **mm/mmHg)**	4.07 (2.28–7.44)	6.40 (3.08–10.75)	0.008
**AD (10** ^**-3**^ **mmHg** ^**−1**^ **)**	3.08 (1.57–5.26)	5.33 (2.80–9.79)	< 0.001
**AS (%)**	8.21 (4.24–13.07)	10.66 (6.01–18.23)	0.039
**ASI**	6.10 (3.64–12.47)	3.79 (2.40–8.50)	0.003
**AEM (10** ^**-1**^ **Kpa)**	8.67 (5.08–16.97)	5.00 (2.72–9.53)	< 0.001

The values are presented as the means ± standard deviations or medians [Q25—Q75]. T2DM: Type 2 diabetes mellitus; AoS: aortic end-systolic diameter; AoD: aortic end-diastolic diameter; AC: aortic compliance; AD: aortic distensibility; AS: aortic strain; ASI: aortic stiffness index; AEM: Aortic Peterson’s elastic modulus

### Correlations between indices of aortic elasticity and relevant variables

**Fig 4:**
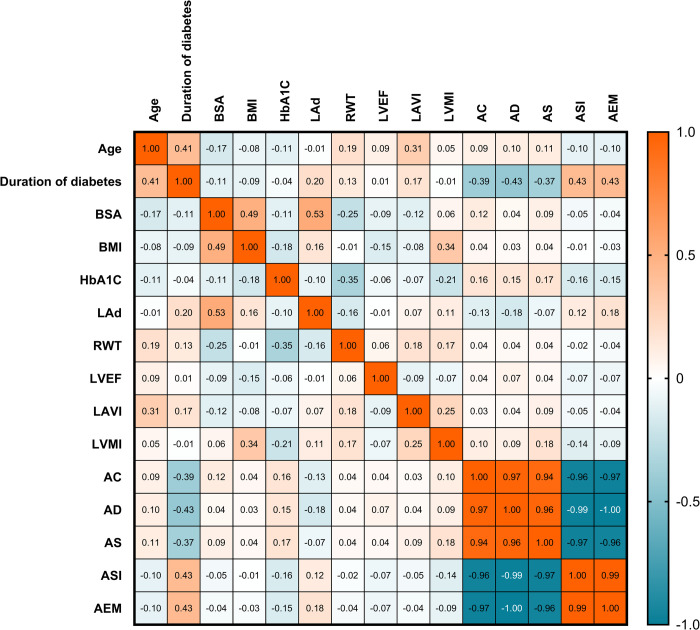
Correlations between anthropometric and echocardiographic features and computed aortic indices. BSA: body surface area; BMI: body mass index; HbA1c: glycated hemoglobin; LAd: left atrial diameter; RWT: relative wall thickness; LVEF: left ventricular ejection fraction; LVMI: left ventricular mass index; AC: aortic compliance; AD: aortic distensibility; AS: aortic strain; ASI: aortic stiffness index; AEM: Aortic Peterson’s elastic modulus.

An analysis was performed to explore the relationships between anthropometric and echocardiographic features and computed aortic indices. **Fig 4** visually illustrates the results in a heatmap. The results revealed that in the T2DM group, the duration of diabetes was associated with arterial elasticity indicators. A longer duration of diabetes correlated positively with greater ASI and AEM, with Spearman’s correlation coefficients of 0.43 for both. In contrast, AC, AD, and AS tended to decrease as diabetes duration increased (Spearman’s correlation coefficients of -0.39, -0.43, and -0.37, respectively).

### Elevated aortic elasticity indices independent of blood pressure

**Table 3** indicates that T2DM remains significantly associated with alterations in aortic elasticity measures after adjusting for both systolic and diastolic blood pressure. Specifically, T2DM is linked to a significant decrease in the AC measure (β = -0.022, p = 0.029) and AD measure (β = -3.077, p < 0.001), suggesting that the reduced elasticity in these parameters is independent of blood pressure. Conversely, T2DM is associated with a significant increase in the ASI (β = 3.335, p = 0.028) and AEM measure (β = 4.788, p = 0.030), indicating that these increases in ASI and AEM are independent of blood pressure effects. Although the AS shows a trend toward reduction (β = -3.259), this result is not statistically significant (p = 0.092), suggesting that the relationship between T2DM and AS may be more complex or less robust after accounting for blood pressure.

**Table 3:** Generalized linear model results of aortic elasticity indices adjusted by systolic and diastolic blood pressure.

Arterial elastic indices	Coefficient (β)	SE	Z value	P value	95% CI
**AC (10** ^**-2**^ **mm/mmHg)**	-0.022	0.010	-2.219	0.029	[-0.042, -0.003]
**AD (10** ^**-3**^ **mmHg** ^**−1**^ **)**	-3.077	0.896	-3.433	<0.001	[-4.834, -1.320]
**AS (%)**	-3.259	1.918	-1.699	0.092	[-7.019, 0.500]
**ASI**	3.335	1.498	2.226	0.028	[0.398, 6.272]
**AEM (10** ^**-1**^ **Kpa)**	4.788	2.179	2.197	0.030	[0.516, 9.059]

T2DM: Type 2 diabetes mellitus; AoS: aortic end-systolic diameter; AoD: aortic end-diastolic diameter; AC: aortic compliance; AD: aortic distensibility; AS: aortic strain; ASI: aortic stiffness index; AEM: Aortic Peterson’s elastic modulus; CI: Confidence Interval; SE: Standard Error.

## Discussion

There is growing interest in studying the association between aortic flexibility and cardiovascular disease. There are various methods available for assessing arterial elasticity. Among the wide range of choices, M-mode echocardiography remains effective at capturing the motions and functions of the heart and surrounding structures, including assessing the ascending aorta. Despite the better temporal resolution of M-mode echocardiography, it is still being disregarded in international guidelines [33]. Substantial efforts have been made to investigate the relationships between insulin resistance (and T2DM) and aortic elastic characteristics. This study was performed in Vietnam with a group of T2DM patients from low- and middle-income countries to assess the feasibility and cost-effectiveness of M-mode echocardiography in daily practice.

### Comparison of aortic elasticity indices between patients with T2DM and healthy controls

It is generally known that vascular elasticity often begins to decline in middle age and worsens over time [30]. Throughout the aging process, the elasticity of the endothelium layer gradually decreases, which significantly impacts the arteries’s structure due to reduced elastic fiber activity, particularly in major arteries such as the aorta [34]. The deterioration of arterial elasticity is caused by a variety of mechanisms, including the development of endothelial dysfunction and the impairment of vasodilation mechanisms via endothelium-derived nitric oxide (NO) and the absence of ETB receptors, which increase the plasma endothelin-1 concentration because these receptors contribute to endothelin-1 destruction [35, 36].

In this study, we measured the elasticity of the aorta using five indices: AC, AD, ASI, AS, and AEM. The T2DM group had significantly lower AC, AD, and AS indices than the comparison group but showed higher ASI and AEM. Various other studies have also confirmed changes in aortic stiffness in patients with diabetes compared to healthy individuals. For instance, Hala et al. demonstrated that patients with T2DM exhibit increased aortic stiffness compared to healthy participants, even in the absence of hypertension or cardiovascular complications [37]. A prospective analysis by Elias et al. comprising 508 community-dwelling participants in New York also indicated that T2DM, particularly when poorly controlled, is significantly associated with a higher risk of arterial stiffness later in life [38]. Additionally, Aslan’s study found that even in patients with prediabetes, alterations in aortic stiffness are evident, with aortic strain and distensibility being lower compared to healthy adults [17].

Despite significant breakthroughs in T2DM treatment, the risk of cardiovascular morbidity and mortality continues to increase [39]. Recent research has revealed a complex link between cardiovascular mortality risk and T2DM, which is thought to be caused in part by increased arterial stiffness [40]. The literature has shown that people with T2DM have a much greater incidence of increased arterial stiffness than individuals without diabetes [41]. This can be attributed to insulin resistance and hyperglycemia. These conditions activate the renin-angiotensin-aldosterone system and enhance the expression of type 1 angiotensin receptors in vascular tissue, leading to arterial wall hypertrophy and fibrosis [42–46]. Increased arterial stiffness causes irreversible pathogenic loads, particularly in patients with diabetes. A persistent increase in arterial stiffness results in increased transfer of pulse pressure into the microcirculation, which is directly associated with a greater risk of disorders such as hypertension, chronic renal disease, coronary artery disease, heart failure, and stroke [47–50]. Our study revealed substantial differences in AC, AD, AS, ASI, and AEM between individuals with T2DM and individuals without diabetes, which suggests further screenings and interventions for these patients are needed for better outcomes.

### Correlations between aortic elasticity indices and diabetic conditions

Our study did not identify any correlation between the HbA1c level and aortic elasticity indices (**Fig 3**).

The relationship between HbA1c, a long-term blood glucose control marker, and aortic stiffness remains inconsistent across various studies. For instance, Martagón AJ et al. found that HbA1c was significantly associated with arterial stiffness only in diabetic patients with hypertension. In contrast, in those without hypertension, arterial stiffness correlated with the duration of diabetes rather than HbA1c levels [51]. Similarly, Chen Y et al. reported only a weak association between HbA1c and aortic elasticity [52]. This inconsistency may be attributed to the nature of HbA1c measurements, which reflect average blood glucose levels over the preceding three months rather than offering a complete picture of long-term glucose control, where factors like time in range, fasting plasma glucose, or postprandial plasma glucose also play crucial roles [53]. As a result, HbA1c may not fully capture the gradual progression of arterial stiffness, which develops over an extended period.

Emerging evidence suggests that long-term glucose variability, assessed as visit-to-visit variability of HbA1c, could significantly impact on aortic stiffening. A study by Fang Q et al., involving 2,115 patients with type 2 diabetes in China, indicated that long-term visit-to-visit HbA1c variability was independently associated with the progression of aortic stiffness. The study highlighted that HbA1c variability strongly predicts subclinical atherosclerosis in individuals with type 2 diabetes [54]. Additionally, research by Ceriello A et al., involving 101,533 people with type 2 diabetes without cardiovascular diseases, demonstrated that the correlation between HbA1c variability and the development of cardiovascular complications was evident regardless of the HbA1c level. Even when HbA1c remained within the target range during follow-up, the risk associated with HbA1c variability was higher for primary outcomes, expanded secondary outcomes, and stroke [55].Therefore, emphasizing the control of long-term blood glucose fluctuations is crucial in comprehensive diabetes management strategies to reduce aortic stiffening and improve cardiovascular health outcomes among individuals with diabetes.

Nevertheless, we did find significant correlations between the duration of diabetes and arterial elasticity indices, as shown in the heatmap (**Fig 3**). A longer duration of diabetes is linked to greater stiffness, whereas elasticity decreases accordingly. A study by Małgorzata Dec-Gilowska et al. demonstrated that the ASI was significantly elevated in patients with T2DM lasting more than 7 years compared to those with a duration of less than 7 years [18]. The study by Agnoletti et al. found that as the duration of diabetes increases, aortic stiffness also rises and that diabetes duration is an independent determinant of aortic stiffness in individuals with type 2 diabetes [56].

In diabetes, the duration of T2DM is intricately linked to the progression of disease complications [9]. Agnoletti et al. also demonstrated that the duration of diabetes correlates with microvascular complications, regardless of renal function, and is associated with macrovascular complications due to increased aortic stiffness. Their comprehensive analysis underscores the impact of prolonged diabetes on vascular health, highlighting the significance of managing aortic stiffness to mitigate these risks [56]. By recognizing the pivotal role of diabetes duration in the progression of arterial stiffness, early diagnosis, and proactive treatment become paramount in optimizing patient care and outcomes.

### Limitations of the study

The single-center design and limited sample size constrain our study. The small sample size is restrictive, potentially introducing bias and diminishing statistical power. Therefore, there is a need for multicenter research with a larger sample size to obtain more vital clinical evidence. Additionally, our study is only a cross-sectional investigation that can demonstrate associations but not causal relationships. Hence, longitudinal studies are required to address this issue. Moreover, arterial stiffness parameters were measured in the central aorta, whereas blood pressure was measured in peripheral arteries. However, due to ethical considerations, our study solely relied on peripheral blood pressure measurements instead of invasive arterial pressure measurements. To address the potential discrepancies between central and peripheral blood pressure measurements, future studies could incorporate non-invasive methods to estimate central blood pressure, such as pulse wave analysis or the use of transfer functions. These methods would allow for a more accurate assessment of the relationship between arterial stiffness and central blood pressure without the need for invasive procedures. Furthermore, validating these non-invasive methods against central pressure measurements in a subset of patients could further strengthen the findings and provide more comprehensive insights into arterial stiffness in the studied population. Another limitation of this study is that M-mode echocardiography cannot measure axial elongation of the ascending aorta, as it cannot assess deformations in the axial direction using anatomical landmarks such as the aortic root or arch bifurcations. Advanced imaging modalities like computed tomography and magnetic resonance imaging are required to comprehensively evaluate circumferential and axial deformations. Future studies aiming for a deeper assessment of aortic mechanics should consider incorporating computed tomography or magnetic resonance imaging. Additionally, given the circumstances at our research site, where alterations in medication regimens are common, our study did not assess the impact of different drug treatments on arterial stiffness. Consequently, more significant and longer-term studies are needed to evaluate this aspect.

## Conclusions

M-mode echocardiography detected a notable difference in aortic elasticity indices was detected between the T2DM patient group and the control group.

## Supporting information

S1 Graphical abstract (TIF)
