# Correction: Alterations in aortic elasticity indices among type 2 diabetes patients in a low and middle income country using M-mode echocardiography: A cross-sectional comparative study

**DOI:** 10.1371/journal.pone.0321115

**Published:** 2025-03-25

**Authors:** Hai Nguyen Ngoc Dang, Thang Viet Luong, Quan Nguyen Khoi, Uyen Ngoc Phuong Nguyen, Nguyen Nguyen Khoi Pham, Hieu Thi Nguyen Tran, Hung Khanh Tran, Mai Thi Thu Cao, Binh Anh Ho, Thang Chi Doan, Hung Minh Nguyen, Tien Anh Hoang, Minh Van Huynh

The ninth author, Binh Anh Ho, is incorrectly noted as the corresponding author. The correct corresponding author is Tien Anh Hoang. Tien Anh Hoang ‘s email address is: hatien@huemed-univ.edu.vn, hatien@hueuni.edu.vn.
