# Supplementary figures and images for: Alterations in aortic elasticity indices among type 2 diabetes patients in a low and middle income country using M-mode echocardiography: A cross-sectional comparative study
Source: PLoS One. 2024 Oct 24;19(10):e0305799. doi: 10.1371/journal.pone.0305799 (PMC11500911; doi:10.1371/journal.pone.0305799)

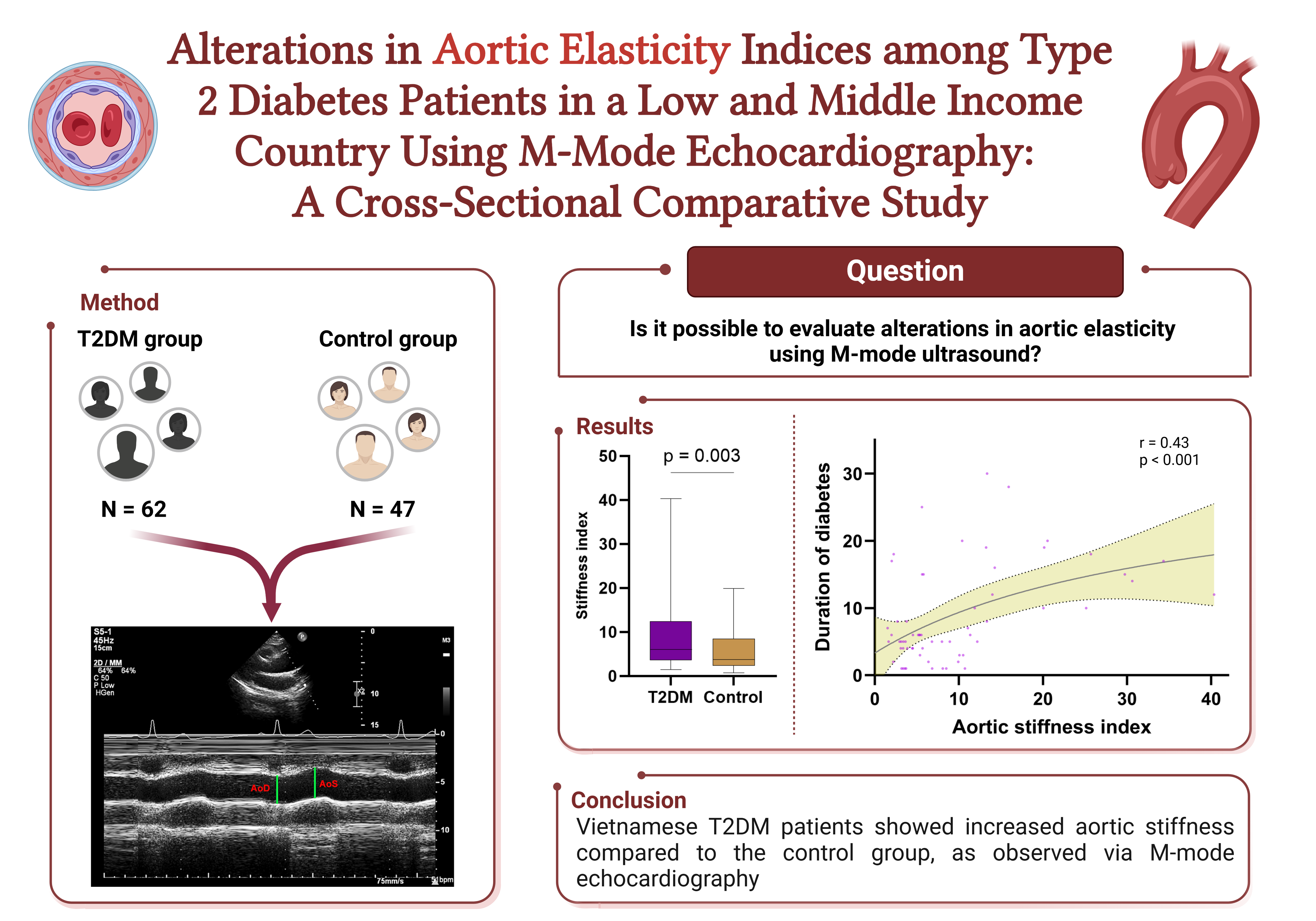

Supplement: S1 Graphical abstract — (TIF) [file pone.0305799.s001.tif]
